# Subchondral bone histology and grading in osteoarthritis

**DOI:** 10.1371/journal.pone.0173726

**Published:** 2017-03-20

**Authors:** Olli-Matti Aho, Mikko Finnilä, Jerome Thevenot, Simo Saarakkala, Petri Lehenkari

**Affiliations:** 1 Department of Anatomy and Cell Biology, Institute of Biomedicine, University of Oulu, Oulu, Finland; 2 Research Unit of Medical Imaging, Physics and Technology, Faculty of Medicine, University of Oulu, Oulu, Finland; 3 Department of Diagnostic Radiology, Medical Research Center, Oulu University Hospital, Oulu, Finland; 4 Division of Orthopaedic and Trauma Surgery, Department of Surgery, Medical Research Center, Oulu University Hospital, Oulu, Finland; Universite de Lyon, FRANCE

## Abstract

**Objective:**

Osteoarthritis (OA) has often regarded as a disease of articular cartilage only. New evidence has shifted the paradigm towards a system biology approach, where also the surrounding tissue, especially bone is studied more vigorously. However, the histological features of subchondral bone are only poorly characterized in current histological grading scales of OA. The aim of this study is to specifically characterize histological changes occurring in subchondral bone at different stages of OA and propose a simple grading system for them.

**Design:**

20 patients undergoing total knee replacement surgery were randomly selected for the study and series of osteochondral samples were harvested from the tibial plateaus for histological analysis. Cartilage degeneration was assessed using the standardized OARSI grading system, while a novel four-stage grading system was developed to illustrate the changes in subchondral bone. Subchondral bone histology was further quantitatively analyzed by measuring the thickness of uncalcified and calcified cartilage as well as subchondral bone plate. Furthermore, internal structure of calcified cartilage-bone interface was characterized utilizing local binary patterns (LBP) based method.

**Results:**

The histological appearance of subchondral bone changed drastically in correlation with the OARSI grading of cartilage degeneration. As the cartilage layer thickness decreases the subchondral plate thickness and disorientation, as measured with LBP, increases. Calcified cartilage thickness was highest in samples with moderate OA.

**Conclusion:**

The proposed grading system for subchondral bone has significant relationship with the corresponding OARSI grading for cartilage. Our results suggest that subchondral bone remodeling is a fundamental factor already in early stages of cartilage degeneration.

## Introduction

Osteoarthritis (OA) is a progressive joint disorder characterized by the uneven and gradual degeneration of articular cartilage, joint pain, stiffness and loss of function in the absence of chronic autoimmune or autoinflammatory mechanisms [1]. It is the most common form of arthritis and a significant factor in the public health of industrialized countries [2]. In the United States alone, over 27 million adults are affected by OA and its prelevance is rising all the time [3]. OA has traditionally been seen primarily as a disorder of the articular cartilage [4]. However, increasing evidence suggests that OA should be considered a disease of the whole joint unit and a gradual shift from cartilage-centered view towards a system biology approach can be seen [1–2, 4–7].

The main methods of OA diagnostics are clinical evaluation by a physician, followed by radiography, magnetic resonance imaging (MRI), and in selected cases, arthroscopy [8]. Diagnosis of OA is still mainly focused on detecting cartilage degeneration and skeletal changes are acknowledged only at later stages, even in histological assessment. The first widely accepted and adopted histological OA grading system was the Mankin scoring [9]. It focuses on architectural changes in articular cartilage and tidemark while also attending to cellular changes and proteoglycan content of cartilage [9–10]. A more novel, and presumably more consistent [10–11], Osteoarthritis Research Society International (OARSI) grading system focuses on the depth of cartilage degeneration and its extent over the whole joint surface [12]. OARSI grading system also accounts for some subchondral bone changes in the higher grades. Despite its growing popularity, OARSI grading system is yet to be systematically compared with the changes in subchondral bone, especially in lower OARSI grades.

Regardless of their omission in common histological evaluation, the changes that occur in subchondral bone in osteoarthritic joints have been widely recognized in literature [1, 13–15]. In early OA, the mineral apposition rate of subchondral bone and bone remodeling increase and new remodeling sites arise, which leads to reduced thickness of the subchondral plate [16]. As degeneration advances, changes in remodeling balance occur in four main processes: reduced bone turnover, subchondral sclerosis, thickening of calcified cartilage and thinning of trabeculae [17]. The defining feature of subchondral bone in late-stage OA is clearly increased bone volume and apparent density [18].

Bobinac *et al*. [19] previously compared articular cartilage and subchondral bone histomorphometry in humans utilizing Mankin score. Using four histomorphometric parameters (bone volume, trabecular thickness, trabecular number and trabecular separation) they found significant linear correlations between articular cartilage degeneration and subchondral bone changes. Bone volume and trabecular thickness were found to increase, whereas trabecular number and separation decreased. They also suggested the changes of subchondral bone to be secondary to cartilage damage. To our knowledge, there are no studies in which the subchondral bone histological changes have been directly correlated with OARSI grading system. Furthermore, no literature can be found aiming to incorporate subchondral bone into complete histological assessment of OA.

We believe that the subchondral bone should not be disregarded in histological grading of OA. There is indisputable evidence [1, 13–19] that subchondral bone remodeling is an essential part of OA pathogenesis but much less is known about the causality. Much like current histological cartilage OA grading scales (Mankin, OARSI), a standardized and easily adaptable scale for subchondral bone would make evaluating and reporting of histological data more consistent. Furthermore it could help us to link the changes in cartilage and bone for a better understanding of joint degeneration. Ultimately we believe that the histological features both of cartilage and bone should be incorporated into one standardized grading scale.

In the present study, based on previous literature, we aimed to create a simple proposal of a histological grading system for changes occurring in subchondral bone during OA. This new grading system was correlated with the OARSI grading of overlying cartilage. Furthermore, subchondral bone histological changes were specifically analyzed quantitatively with digital image analysis software by measuring the thickness of non-calcified and calcified cartilage as well as subchondral plate. Finally, the internal structure of cartilage-bone interface was analyzed using the local binary patterns (LBP) based method.

## Materials & methods

### Selection of patients and sample processing

20 patients (mean age 74.5 years, range 57-88 years) suffering from OA and undergoing uni- or bilateral total knee arthroplasty in Oulu University Hospital were randomly selected for this study (Table 1). Tibial plateau surface, which is routinely extracted during the surgery, was collected and frozen to serve as a sample material. Collection of tissue samples for this study was authorized by the institutional review board of The Northern Ostrobothnia Hospital District (EETTMK: 191/2001) and a written consent was obtained from each patient in this study. Each tibial plateau surface was visually graded into three categories in terms of uniformity of the articular cartilage: 1) most inviolable (intact) cartilage, 2) moderate cartilage degeneration, and 3) partly or fully exposed subchondral bone. Grading was conducted by an experienced orthopaedist (PL). Using an industrial grade upright drill (TA-FE Drill Press model TFL-16, Teräs Oy, Finland), three adjacent osteochondral sample series, one sample (diameter 6mm) from each of the three categories of each plateau, were extracted with special care to avoid any cartilage damage. Two series were used for histology and were formalin fixed, decalcified and paraffin embedded and finally 5 μm thick sections were stained with Safranin O or hematoxylin and eosin stain. Decalcification was done using 14% EDTA solution for four weeks for paraffin embedded samples. One series was used for non-decalcified histology. These ’hard tissue’ samples were prepared using ethanol series-dehydrated samples and methacrylate based resin including dibutyl phthalate, benzoylperoxide in 30°C for 2 days and at 36°C for a 5-7 days. PMMA sections were cut into 5 μm thickness and after PMMA removal with 2-Mehoxyethyl acetate the sections were stained with Masson Trichrome method.

**Table 1:** Patient characteristics.

Patient number	Age (years)	Sex	BMI (kg/m^2^)	Rheumatoid arthritis	Mean OARSI Score	Operated knee
1	57	Female	35.6	-	3.4	Both
2	61	Female	36.1	-	3.7	Left
3	79	Male	27.8	-	4.3	Right
4	87	Female	22.3	-	5.1	Right
5	78	Female	27.6	*	4.2	Left
6	83	Female	26.5	-	4.3	Right
7	78	Female	28.7	-	3.9	Left
8	88	Female	30.2	-	3.8	Right
9	84	Female	25.0	-	4.4	Right
10	76	Female	25.9	-	3.6	Right (medial)
11	84	Female	34.4	-	4.2	Left
12	77	Male	26.0	-	4	Right
13	66	Female	26.2	-	4.1	Right
14	61	Female	33.3	-	4.4	Both
15	75	Female	34.1	-	4.6	Both
16	62	Female	30.2	-	4.7	Left
17	61	Female	33.3	-	4	Both
18	81	Female	34.6	+	3.4	Right
19	70	Male	36.1	-	3.7	Left
20	82	Female	30.4	-	3.6	Right

BMI = Body mass index.

* = patient 5 suffered from spondylarthritis. Only medial tibia of patient 10 was operated.

+ = yes

- = no.

Mean OARSI score from all the samples collected from each patient is presented.

### OARSI grading

Standardized OARSI grading system [12] was used to assess the grade of OA in histological sections. Three independent researchers (OMA, MF, SS), familiar with OARSI grading, assessed Safranin O stained samples accordingly.

### Subchondral bone grading

**Fig 1:**
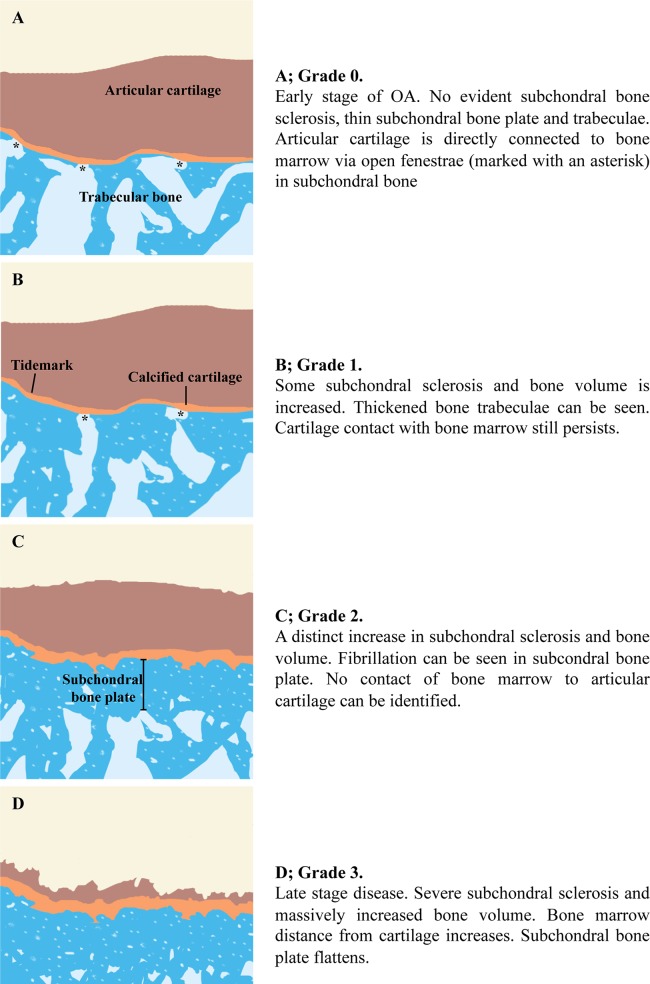
Subchondral bone changes and grades. (A) Grade 0 Early stage of OA. No evident subchondral bone sclerosis, thin subchondral bone plate and trabeculae. Articular cartilage is directly connected to bone marrow via open fenestrae (marked with an asterisk) in subchondral bone. (B) Grade 1. Some subchondral sclerosis and bone volume is increased. Thickened bone trabeculae can be seen. Cartilage contact with bone marrow still persists. (C) Grade 2. A distinct increase in subchondral sclerosis and bone volume. Fibrillation can be seen in subcondral bone plate. No contact of bone marrow to articular cartilage can be identified. (D) Grade 3. Late stage disease. Severe subchondral sclerosis and massively increased bone volume. Bone marrow distance from cartilage increases. Subchondral bone plate flattens.

**Fig 2:**
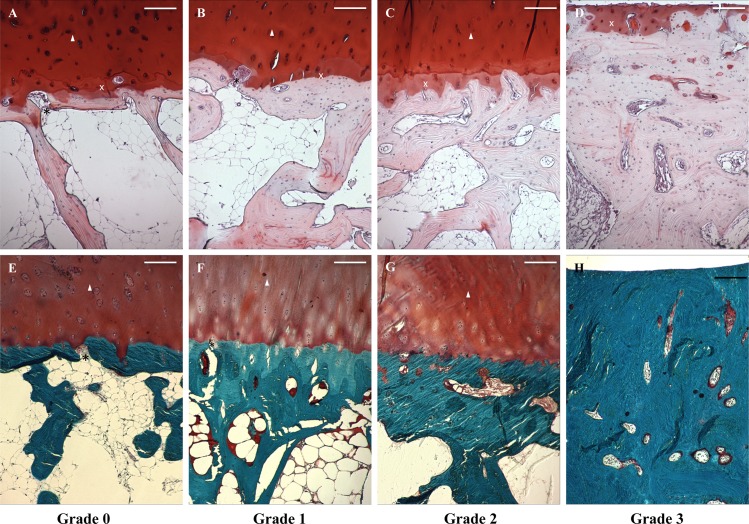
Safranin O (A-D) and Masson’s trichrome stained histological samples of subchondral bone grades. Images taken with a light microscope using digital camera. White triangle marks articular cartilage, white cross shows calcified cartilage. (A and E) Black asterisks marks fenestrae in subchondral bone plate connecting the articular cartilage to bone marrow in grade 0 and (B and F) grade 1. (C and G) Fibrillation on subchondral bone plate can be seen in grade 2. (D and H) Distinctive sclerosis and loss of articular cartilage mark late-stage OA in grade 3. Scale bar 200 μm.

Based on previous studies describing the changes in subchondral bone [1, 13–18] and the rough observations of OA sample material by two researchers (OMA, PL), a simple four-stage grading system for subchondral bone (Figs 1 and 2) was proposed.

*Grade 0* represents the most inviolable subchondral bone or very early OA changes. No subchondral sclerosis is evident and subchondral plate is thin. Articular cartilage is directly connected to bone marrow via open fenestrae in subchondral plate.

*Grade 1* presents mild subchondral sclerosis and an increase in bone volume. Open subchonral bone fenestrae connecting bone marrow to articular cartilage still exist. Thickened subchondral bone trabeculae can be seen. Some architectural changes can be seen in subchondral plate.

*Grade 2* is characterized by a distinct increase in subchondral bone sclerosis and volume. Fibrillation in subchondral bone plate can be seen. No open connection between bone marrow and cartilage can be identified.

*Grade 3* represents the late-stages of OA. Severe sclerosis and a massive increase in subchondral bone volume are the typical features. Loss of articular cartilage and flattened subchondral plate can be also seen.

Similarly to OARSI grading, three independent researchers (OMA, MF, SS) assessed the subchondral bone grade after being introduced and familiarized with the new grading system.

### Image analysis

Quantitative image analysis was conducted under light microscopy (Nikon Eclipse; Nikon Instruments, Japan) using x5 original magnification, digital camera and digital image analysis software (MCID-M5+; Imaging research, Canada). Thickness of uncalcified cartilage, calcified cartilage and subchondral bone was measured from five different randomly selected but evenly distributed points within one microscope view.

### Segmentation of the cartilage-bone interface

**Fig 3:**
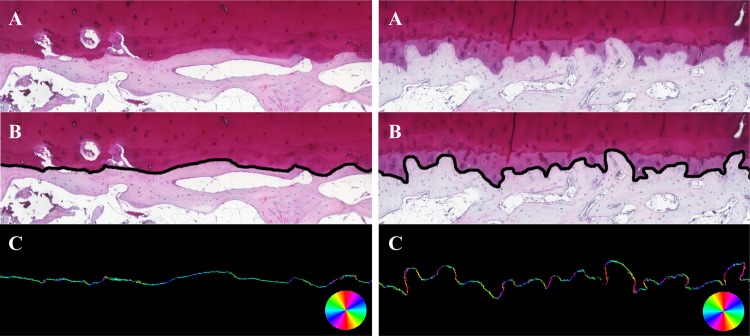
LBP analysis of the interface bone-cartilage for a sample with early OA. On left an image of a sample with light degeneration (OARSI: 1.0) and a sample with severe OA on right (OARSI: 4.5). (A) Original image, original magnification x5. (B) Manual segmentation of the interface bone-cartilage and (C) distribution of local angles at the interface from LBP analysis.

The cartilage-bone interface (BCI) is important for the proposed grading system, it was specifically analysed for the samples with Safranin-O stain (magnification x5, Fig 3) and with remaining cartilage (OARSI<5, N = 18). Shape and structure of BCI was correlated to OARSI and proposed grading system. For this purpose, the interface was first manually segmented in each picture to select only the data not affected by artefacts (e.g. cuts in the samples, blurry and out of focus areas) and containing BCI in the middle of a seven pixels thick layer (Fig 3). Subsequently, a custom-made algorithm developed in MATLAB (version R2014b, MathWorks, Inc., Natick, MA, USA) was applied to keep solely the pixels within this layer that are either at the interface itself or at its edges. As a result, the final mask for analysis of the interface was a thinner layer (≈three pixels thick) than the original manually segmented data.

### Local binary patterns (LBP) based method to assess local angles

To assess the local angles within the pixels of the BCI, LBP-based method was adapted from the previous literature [20–21]. Briefly, the neighbours of a central (studied) pixel are evaluated to assess if some have an equal or higher grey level values than the studied pixel. Based on the location of these occurrences, a local pattern is derived, providing information on the local orientation of the neighbourhood. Eventually, the result was the distribution of local angles distribution of BCI (Fig 3), describing the internal structure or “fibrillation” of the BCI.

### Cartilage-bone interface parameters

For each sample, both the entropy and the homogeneity of the local angles were calculated. The entropy of local angles describes the randomness of local angles. The homogeneity index of the angles describes spatial distribution of the elevation angles and provides indication on its continuity. An angle-level co-occurrence matrix (ALCM) was first calculated with volumetric rotational invariance, to assess the spatial distribution of the elevation angles. Eventually in this study, a high value of homogeneity of the angles corresponds to an interface made of consecutive pixels with similar elevation angles, while a low value suggests the occurrence of sharp angles along the BCI.

### Statistical analysis

Statistical analysis was performed with the Statistical Package for the Social Sciences (SPSS) version 20.0 (Chicago, Illinois, USA). The intra-observer repeatability and consistency of the proposed subchondral bone grading system was analyzed by calculating the intra-class correlation coefficient (ICC). One-way ANOVA and Turkey’s post hoc test were used for studying the relationship between OARSI grading and subchondral bone grading, as well as between subchondral bone grading and image analysis data. As parameters were applicable, independent t-test between OA samples grouped by severity assessed from the subchondral bone was applied to analyse how well the LBP-based parameters are distributed in this grading system. Linear regression analysis was performed between LBP-based parameters and OARSI grades.

## Results

Osteochondral samples from the tibial plateau had a large internal variation in the OARSI grade depending on the harvesting site. Visually most inviolable graded samples had a mean OARSI score of 2.1, whereas visual grade 2 and 3 reached mean scores of 4.4 and 5.3, respectively. Overall, the OARSI grades varied from 0.5 to 6 (mean±SD = 4.07±1.31) and subchondral bone grades varied from 0 to 3 (mean±SD = 1.94±0.99). The intra-observer reliability of subchondral bone grading was excellent ICC = 0.97.

### General histological features of subchondral bone

**Fig 4:**
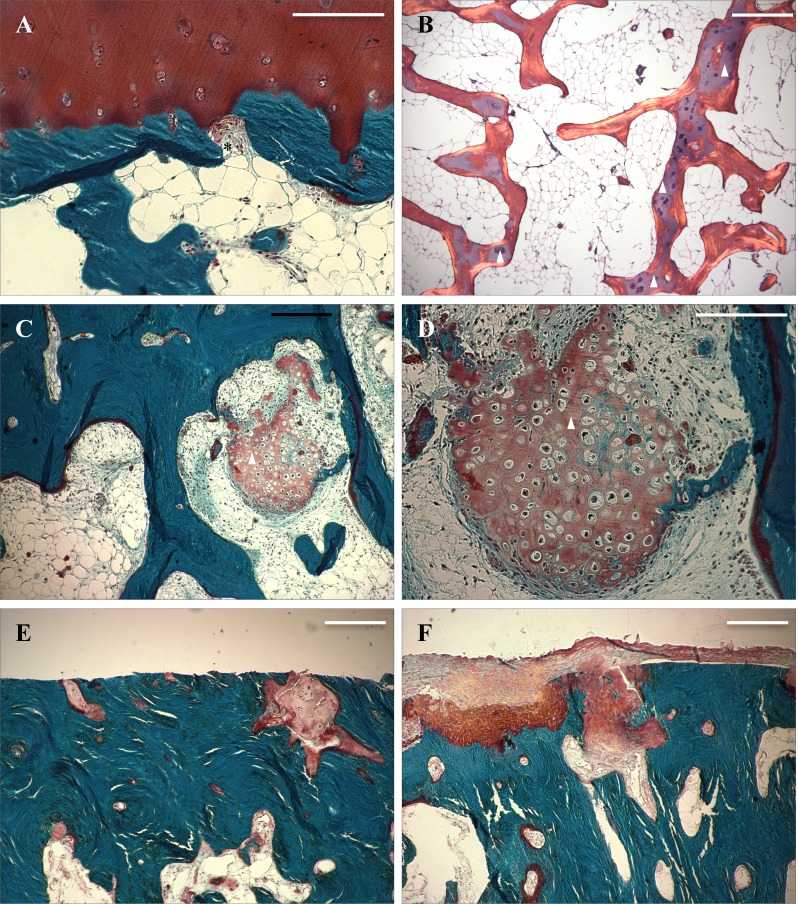
Masson’s trichrome (A, C-F) and HE stained (B) histological images of the features of subchondral bone. (A) Subchondral bone fenestra marked with an asterisk. (B) Subchondral trabeculae showing some intra-trabecular cartilage (triangles), polarized light microscopy. (C-D) Some of the more degenerated samples were seen with cartilage (triangle) among trabecular bone. (E-F) Late-stage degeneration samples revealing degenerated and apoptotic cartilage. Scale bar 200 μm.

Subchondral bone histology undergoes distinct changes in OA and increasing thickness of bone plate can clearly be seen. The surface of subchondral plate is also altered as more rugged and uneven fibrillating surface pattern can be seen in samples with more advanced joint degeneration. Similarly, trabecular bone structure seems to become disoriented. Late-stage OA samples typically showed pronounced cartilage erosion accompanied by flattened subchondral plate and massive subchondral sclerosis (Figs 2 and 4).

### Subchondral bone grading was in accordance with OARSI grades

**Fig 5:**
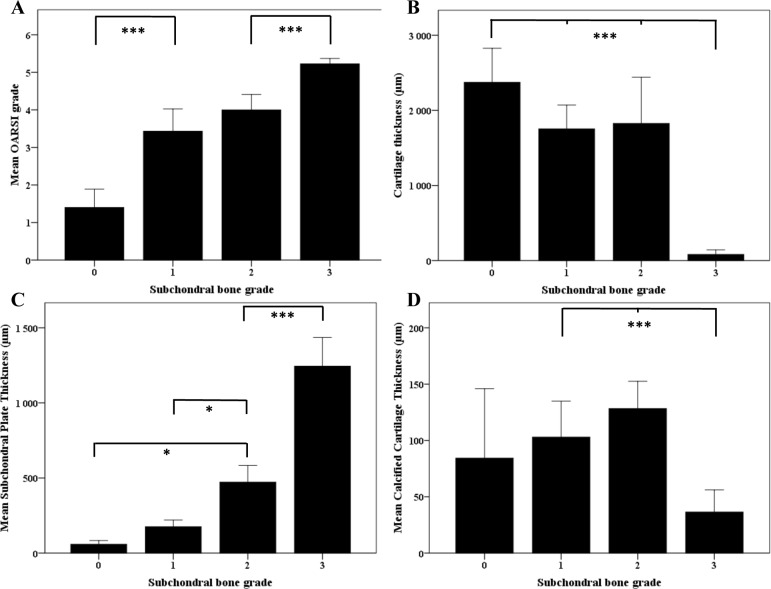
Mean OARSI scores of subchondral bone grades and histological image analysis data in correlation to samples bone OA grade. (A) OARSI score increases by subchondral oa grade. Samples with subchondral grade 0 reached mean OARSI score of 1.46. Subchondral OA grades 1, 2 and 3 had mean OARSI scores of 3.45, 3.99 and 5.19 respectively. Each bar and point represents the mean ± S.E. (B) Mean cartilage thickness. (C) Mean subchondral bone plate thickness. (D) Mean calcified cartilage thickness. Each bar and point represents the mean ± S.E. * p<0.05, *** p<0.001

Samples with subchondral bone grade 0 had a mean OARSI grade of 1.46, whereas samples with subchondral grades 1,2 and 3 had mean OARSI grades of 3.45; 3,99 and 5.19, respectively (Fig 5A).

### The thickness of articular cartilage decreases in samples with higher subchondral bone grade

Samples with subchondral bone grade 0 had the highest cartilage thickness (mean±SD = 2374 μm±506) and lowest calcified cartilage thickness (mean±SD = 84 μm±69). However, no major differences in cartilage layer was observed in samples between subchondral bone grade 1 (mean±SD = 1753 μm±615) and 2 (mean±SD = 1826 μm±1268). Grade 2 samples had thicker calcified cartilage layer (mean±SD = 128 μm±50) than grade 1 (mean±SD = 104 μm±62,44) but the difference was not statistically significant. Subchondral bone grade 3 samples had already lost most of the cartilage and calcified cartilage layers (mean±SD = 82 μm±142 and 36 μm±46, respectively). (Fig 5B and 5D).

### Subchondral plate thickness greatly increases by subchondral bone grade

Grade 0 samples had the lowest subchondral bone plate thickness (mean±SD = 58 μm±28). The thickness increased 3-fold already in grade 1 (mean±SD = 176 μm±87), increased up to 8-fold in grade 2 (mean±SD = 473 μm±229) and ultimately to over 21-fold in grade 3 (mean±SD = 1245 μm±448) (Fig 5C).

### Fibrillation in cartilage-bone interface increases in moderate joint degeneration

**Fig 6:**
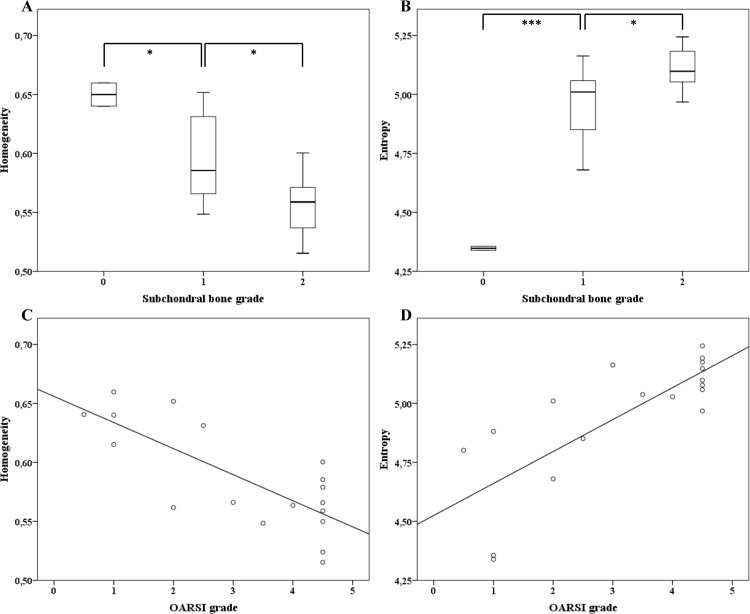
LBP based homogeneity and entropy of subchondral bone in correlation to bone and cartilage OA grade. (A) Homogeneity and (B) entropy in samples with subchondral bone grade 0-2. Grade 3 samples had smooth and worn surface and were excluded. (N = 2, 9 and 7 for grades 0, 1 and 2 respectively). Linear regression models between (C) homogeneity, (D) entropy and cartilage OARSI grade, r^2^(C) = 0.583, r(D) = 0.612. * p<0.05, *** p<0.001.

Cartilage-bone interface plate gets rugged and uneven as OA advances. The LBP-based method to analyze local angles and ‘bone patterns’ was used to quantify this phenomenon. Both the homogeneity and entropy of local angles were able to discriminate the different levels of OA (Fig 6) with statistical significance (*p*<0.05). Homogeneity of the angles was inversely correlated with the OARSI grade (R^2^ = 0.58) while the entropy of angles was positively correlated (R^2^ = 0.61) (Fig 6).

## Discussion

This study focuses on the histomorphometry of subchondral bone in different phases of OA in humans. Based on existing knowledge and our preliminary observations, we proposed a simple four-stage evaluation scale for these changes and tested its correlation to different stages of cartilage erosion.

Cell proliferation and increased matrix remodeling are regarded as the very first signs of OA and both responses can be seen in both the articular cartilage and subchondral bone [1]. Articular chondrocytes try to maintain the integrity and homeostasis of the tissue by synthesizing matrix molecules while they also increase their own destruction with proinflammatory cytokines and destructive enzymes [1]. In parallel to the changes in cartilage, increased cellular activity in the underlying bone ultimately leads to subchondral bone sclerosis with thickening of the cortical plate, extensive remodeling of the trabeculae, and osteophyte formation at the outer edges of the joint [22]. Changes in the subchondral cortical plate and trabeculae are associated with activation of the bone remodeling cycle, most likely as an adaptation to changes in the biomechanics of the joint or in an attempt to repair microdamages [4]. High remodeling in subchondral bone may compromise the biomechanical properties of the tissue [1].

Early OA changes in subcrondral bone are believed to be caused by increased bone remodeling and an increase in remodeling sites within the bone, which further lead to reduced thickness of subchondral plate [13, 23]. In the new grading system, grade 0 represents the samples with most inviolable subchondral bone found in our series of osteoarthritic human tibias. Grade 0 samples are characterized by a thin subchondral plate and trabeculae and a direct connection to bone marrow. The histology of articular cartilage in grade 0 samples did express some mild but evident degeneration having a mean OARSI grade of 1.46. Interestingly, grade 0 samples had a lot of variation in calcified cartilage thickness, which shows heterogeneity among these samples. Thinned subchondral plate and articular cartilage degeneration were associated previously in two different post-traumatic canine OA models [24]. Also in one experimental model with rabbits, increased bone remodeling and thinning of the subchondral plate resulted in increased cartilage damage [25]. Some human studies have also linked increased bone remodeling to progressive OA [26–28].

Increased bone remodeling ultimately leads to increased subchondral bone volume and density which are the definitive features of late-stage OA [18]. Previous studies have also reported both decreased and increased thickness (together with decreased trabecular number and separation) of bone trabeculae, thickened calcified cartilage and reduced bone turnover [17, 19]. A clear increase in subchondral plate thickness is well documented [19, 29–30]. In quantitative analyses on two different human studies, bone mineral density has been reported to increase up to 15% and bone volume up to 30% in OA [30–31]. The mechanical consequences of subchondral sclerosis are, however, not entirely clear [16]. One animal study utilizing sheep with induced subchondral sclerosis (without induced bone remodeling) reported no deterioration of overlaying cartilage even after five years [32] suggesting that subchondral densification alone does not inevitably lead to OA. Furthermore, in another study by Cox *et al*. (2012) noted that in OA the changes in subchondral bone mineralization occur only beneath the areas of significant cartilage destruction [33]. It can be, therefore, suggested that the subchondral sclerosis by itself is not the key factor in OA but rather the increased remodeling as it also causes structural and biochemical changes that affect the functionality of subchondral plate.

The proposed subchondral bone grading scale focuses on bone remodeling, and increase in bone volume is perhaps the most figurative change in higher grades. Subchondral bone grade 1 samples already have increased subchondral plate thickness to some extent and typically an increase in bone trabeculae thickness can be seen. Porous subchondral plate is one fundamental difference from grade 2. Further increase in bone volume and trabecular thickness can be seen in grade 2 and 3 samples, the latter representing late stage disease with massively increased subchondral plate thickness. As in many previous studies [1, 4, 17, 23, 33–34], we found a clear correlation between increased bone remodeling and cartilage degeneration. In addition, measured subchondral bone thickness greatly increased by bone grades. Calcified cartilage thickened up to grade 2 although there was considerable variation. All of our grade 3 samples showed massive cartilage erosion and some had only pieces of calcified cartilage left. The middle grades of our scale (i.e. 1 and 2) had the least differences in cartilage degeneration and average cartilage thickness in grade 2 samples was actually higher than in grade 1 although also showing considerable variation. It can be speculated whether there is “a point of no return” in remodeling response balance somewhere between bone grades 1 and 2 from which onwards the rate of cartilage degeneration increases.

One notable feature of increased subchondral bone remodeling is increased vascularization [13]. One previous study associated elevated angiogenetic factors in synovial fluid with cartilage degeneration [35], and these factors have been shown to increase the synthesis of catabolic enzymes in chondrycytes [34, 36]. Another interesting feature affecting the structural changes in OA is endochondral ossification. In various studies it has been shown that during OA, chondrocytes near the tidemark obtain a hypertrophic phenotype which is typically observed in the growth plate [37]. Chondrocytes start expressing type X collagen and mineralize while vascular endothelian growth factor (VEGF) expression is also increased. Furthermore, this could induce the vascularization observed in OA and be responsible for delivering osteoclast precursors enabling the resorption of mineralized cartilage, similar to endochondral ossification during growth [37]. Endochondral ossification is likely to also affect the architecture of subcondral bone plate during the pathogenesis of OA. In our image analysis, we found some clear arcitechtual changes in subchondral bone plate in samples with distinct degeneration but notable cartilage thickness. In fact, one of the defining features of subcondral bone grade 2 was the increased “fibrillation” in bone surface. In some samples we also witnessed vascular tissue inside the ‘protrusions’ arising from subchondral plate.

In their bone studies, Hirvasniemi *et al*. [20] and Thevenot *et al*. [21] suggested that the entropy of local bone patterns calculated either from radiographs [20] or μCT data [21], is proportionate to the increase of OA severity. Similarly to their results suggesting that “new” local bone patterns occur with an increase of OA level, we report here that the cartilage-bone interface has an increase of “new” local angles with OA. Basically, this increase of entropy suggests that the interface presents more variations in its orientation, going from an almost planar shape to a fluctuating one. The homogeneity of local angles reported here assesses the continuity of the interface, providing an index representative of the sharpness of the curves along the sample. As OA increases, the decrease in homogeneity corresponds to an increase of the interface roughness. The results obtained from both the entropy and homogeneity of local angles suggest that the bone-cartilage interface becomes more chaotic with the increase of OA severity.

Our image analysis revealed also something quite unexpected as in some samples cartilage was found under the subchondral plate. In some cases nodules of hyaline cartilage were found among bone marrow, while some samples had what it seemed like cartilage inside bone trabeculae. We were not able to find any information on such findings in previous literature and the origin of these can be only speculated. Previously, it has been shown that in some cases chronic inflammatory processes can even lead to formation of cartilage among organizing granulation tissue [37]. In fact, one piece of cartilage we found was surrounded by shreds of tissue that shared some similarities with organizing fibrous granulation neotissue. It is known that inflammatory processes have an essential role in the pathogenesis of osteoarthritis and studies have been published on the topic [38].

Only one previous study has systematically compared the histological cartilage grading (Mankin score) with subchondral bone histomorphometry [19]. Subchondral bone volume and trabecular thickness were found to increase in samples with higher level of cartilage degeneration whereas trabecular number and separation decreased. The study classified the samples into three groups or “stages” according to Mankin score each including scores from 0-6, 7-9 and 10-14, respectively. One other study compared joint histology and μCT scans in an experimental rat OA model [39]. The study similarly reported a clear correlation between cartilage degeneration and bone remodeling. In addition, both of these studies concluded that the changes in bone histomorphometric parameters are secondary to cartilage degeneration. This statement is today perhaps somewhat disputed. The grading system we developed did prove itself to be easy-to-apply in histological analysis and intra-observer reliability was excellent. Also when compared to cartilage histology, the bone grading scale does seem quite logical and justifiable it its admittedly robust current form.

The most prominent interfering and limiting factor in our results originates from the understandable limitation of our study and sample collecting methods. Since our patients were selected for joint replacement, the sample population obviously presents various grades of osteoarthritis, although most commonly only the medial sides of the tibial plateus were affected. This results in the lack of intact and ‘truly healthy’ cartilage samples. It has been previously shown that joints with osteoarthritis can have areas of nearly intact and healthy cartilage, and based of our OARSI grading it was very much the case in our samples, too. However, we found no data on whether this is the case for subchondral bone.

## Conclusions

Osteoarthritis causes many structural and biochemical changes in both the articular cartilage and subchondral bone. Whereas these changes have been previously well documented the relation between cartilage and bone degeneration can still be discussed. Increased bone remodeling is however strongly associated with increased cartilage damage. We conclude that subchondral bone should be taken into account in histological assessment of joint degeneration and a more universal grading scale merging cartilage and bone changes for profound histological analysis is needed.

## Supporting information

S1 Dataset Supporting data spreadsheet. (XLSX)
